# Thermal discharge-induced seawater warming alters richness, community composition and interactions of bacterioplankton assemblages in a coastal ecosystem

**DOI:** 10.1038/s41598-021-96969-2

**Published:** 2021-08-30

**Authors:** Meora Rajeev, T. J. Sushmitha, Chairmandurai Aravindraja, Subba Rao Toleti, Shunmugiah Karutha Pandian

**Affiliations:** 1 Department of Biotechnology, Alagappa University, Science Campus, Karaikudi, 630 003 Tamil Nadu India; 2 Department of Periodontology, College of Dentistry, University of Florida, Gainesville, FL USA; 3 Water and Steam Chemistry Division, Bhabha Atomic Research Centre Facilities, Kalpakkam, 603 102 Tamil Nadu India

**Keywords:** Environmental impact, Bacteria, Microbial communities, Environmental microbiology

## Abstract

Despite accumulating evidence on the impact of global climate warming on marine microbes, how increasing seawater temperature influences the marine bacterioplankton communities is elusive. As temperature gradient created by thermal discharges provides a suitable in situ model to study the influence of warming on marine microorganisms, surface seawater were sampled consecutively for one year (September-2016 to August-2017) from the control (unimpacted) and thermal discharge-impacted areas of a coastal power plant, located in India. The bacterioplankton community differences between control (n = 16) and thermal discharge-impacted (n = 26) areas, as investigated using 16S rRNA gene tag sequencing revealed reduced richness and varied community composition at thermal discharge-impacted areas. The relative proportion of *Proteobacteria* was found to be higher (average ~ 15%) while, *Bacteroidetes* was lower (average ~ 10%) at thermal discharge-impacted areas. Intriguingly, thermal discharge-impacted areas were overrepresented by several potential pathogenic bacterial genera (e.g. *Pseudomonas*, *Acinetobacter*, *Sulfitobacter*, *Vibrio*) and other native marine genera (e.g. *Marinobacter*, *Pseudoalteromonas*, *Alteromonas*, *Pseudidiomarina*, *Halomonas*). Further, co-occurrence networks demonstrated that complexity and connectivity of networks were altered in warming condition. Altogether, results indicated that increasing temperature has a profound impact on marine bacterioplankton richness, community composition, and inter-species interactions. Our findings are immensely important in forecasting the consequences of future climate changes especially, ocean warming on marine microbiota.

## Introduction

Coastal marine ecosystems are highly diverse and productive systems on the Earth, providing over US$ 14 trillion worth of ecosystem goods^1^. Among other microorganisms, bacterioplankton community plays a crucial role in maintenance of this ecosystem^2^. Unprecedented reports on bacterioplankton diversity and functionality in this ecosystem provide the testament for conspicuousness as they are involved in several ecologically important services including nutrients recycling, decomposition of organic matter and remineralization^3^. It is well recognized that more than half a fraction of the total carbon flux in marine food web is passed through bacterioplankton^4^. Besides their ecological importance in driving biogeochemical cycles, bacterioplankton communities have amazing capability in responding to environmental perturbations and therefore they are considered as determinants for the ecological status of marine health^5–7^.

The global climate change especially, rising temperature is considered a threat to marine ecosystem, which may influence bacterioplankton activity, diversity and community composition^2^. As per the recent report of the Intergovernmental Panel on Climate Change (IPCC, 2018)^8^, global warming (average surface temperature of the Earth) has raised by approximately 1.0 °C compared to pre-industrial level and increase at the same rate is expected to reach 1.5 °C between 2030 and 2052. Similarly, a model has predicted that global warming is likely to exceed 3 °C by the end of this century^9^. As it is now discernible that rise in global surface temperature is unequivocal, it is essential to decipher the consequences of global warming on bacterioplankton community^10,11^ and ecosystem processes^12^. Though several laboratory manipulation studies have been conducted so far to address the effect of warming on bacterioplankton community, the results are inconsistent. For example, a few studies have shown significant community variations under rising temperature and warming conditions^13–15^, whereas others did not^16,17^. Similarly, number of long-term in situ studies have shown an influence of elevated temperature on bacterioplankton community composition^18,19^.

Elevated seawater temperature due to the thermal discharge of coastal nuclear power plants (NPP) has been recognized as a suitable model to study the response of marine bacterioplankton community to future rise in global temperature^20^. Generally, NPP deploy a large volume of seawater (typically 3 m^3^ per minute per megawatt of electricity generated) from coastal areas as a key source for waste heat rejection^21^. In once-through system, seawater is generally drawn from an intake area, transit through the condenser section for waste heat extraction and then released back to the same coastal region at an outfall area—a location away from the intake point^21^. The discharged warm water that has a temperature of about 7–10 °C above the ambient water causes several adverse impacts on the ecology of receiving water body^22,23^. The adverse effects of thermal discharge on marine ecology was extensively attested by majority of studies, in terms of reduction in fish richness and diversity^24^, enhanced mass mortality in plankton population^22^, growth and development of lower invertebrates^23^, reduction in survival and population density of zooplankton and phytoplankton communities^25,26^, increased virulence of bacterial pathogens^27^, and enhancement in bacterial biofilm formation^28^. However, comparative studies delineating the impact of thermal discharge on bacterioplankton community are rather rudimentary especially by using modern sequencing technologies.

The application of next-generation sequencing (NGS) techniques has enabled the scientific community to explore microbial communities at higher resolution^29^, and thus NGS-based approaches are considered as a reliable tool in assessing integrity and health status of marine ecosystems^30^. To the best of authors’ knowledge, only two studies in China have been conducted so far using NGS technique to elucidate the response of bacterioplankton community to thermal discharges created warming. First study was conducted by Xiong et al.^20^ in the vicinity of Datang coal power plant, and observed that thermal discharge-created elevated temperature stimulates bacterial abundance, grazing rate, and alters the bacterioplankton community structure. Second study examined bacterioplankton community along the thermal flume of Daya Bay and Lingao nuclear power stations during summer and winter seasons and demonstrated a season-specific heterogeneity in bacterioplankton community composition across thermal gradient^31^.

In the light of said reports, we recorded month-wise observational data sets for continuously one year from the vicinity of a coastal NPP located at the south coast of India, Laccadive Sea, Indian Ocean. Unlike previous studies^20,31^ that were conducted to determine bacterioplankton community along the thermal flume, fundamental objectives of our study were to compare bacterioplankton richness and community composition of thermal discharge impacted seawater with unimpacted control. The main questions we aimed to answer are: (i) to what extent thermal discharge-created warming influences the diversity patterns of bacterioplankton, (ii) what are the underlying bacterioplankton groups that constitute microbiota of thermal discharge-impacted and control areas, and (iii) does thermal discharge-created elevation in seawater temperature cause any impact on bacterioplankton co-occurrence network?

## Methods

### Site description and sampling strategy

The field study was conducted in the vicinity of a NPP, located in Kudankulam town (08°10′08′′N, 77°42′45′′E), about 25 km northeast of Kanyakumari at the southern coastal region of India. This area is situated in the distal end of the Gulf of Mannar, endowed with rich marine diversity and has been frequently reported to inhabit various ecosystems such as sandy beaches, coral reefs, rocky shores and mudflats^32^.

Detail description of NPP is given in our previous study^33^. Briefly, with two more units under construction alongside, existing station consisting of two units (Unit-I and -II) of pressurized water reactors (Russian design: VVER-1000/V-412) are currently in operation. Each unit generates 1000 MWe at maximum capacity and employs seawater (from Laccadive Sea) as tertiary coolant at a design flow rate of 75 m^3^ s^−1^. Both units pump seawater from an offshore area (about 1200 m away from the shore) that passes through the condenser and other auxiliary heat exchangers and is discharge back on-shore through two separate outfall structures. The released thermal discharges raise the temperature of receiving water body and form temperature gradient in the nearshore region.

Surface seawater was sampled at monthly interval over one year (12 occasions) spanning from September-2016 to August-2017. The detailed information on seawater sampling and power generating status of both units during the sampling occasions is provided (Supplementary Table S1). A total of nine sampling stations were fixed in the vicinity of NPP by global positioning system; GPS (Supplementary Fig. S1) and monthly cruises were undertaken to collect surface seawater from the same stations during each of the sampling campaigns. Based on the distribution of thermal discharge, these nine sampling stations were categorized mainly into three areas. First area (Station 1–3) is continuously exposed to the thermal discharge of Unit-I and therefore named as outfall area 1 (OA1). Second area (St. 4–6) is located adjacent to the outfall of Unit-II and thus named as outfall area 2 (OA2). Third area (St. 7–9) is located parallel to the seashore at the intake area (IA) and is considered as temperature background area (i.e. a place which is not affected by thermal discharge). In addition to these three areas, three additional stations (St. 10–12) located around 3 km away from the area IA, designated as ambient area (AA) were also sampled during December-2016, March-2017 and May-2017 to compare the seawater in the vicinity of the NPP with that of the absolute ambient seawater. The characters used to define sample names denote months (e.g. September: Sep, October: Oct) and areas (outfall area 1: O1, outfall area 2: O2, intake area: I and ambient area: A) of seawater sampling. Seawater samples were collected in acid-washed and rinsed plastic carboys and were transported to laboratory in ice-cold condition.

### Physicochemical characteristics and nutrients analyses

Seawater physicochemical properties such as temperature, dissolved oxygen (DO) and content of inorganic nutrients such as nitrite, nitrate, ammonium and soluble reactive phosphorus (SRP) were determined as detailed in Supplementary methods.

### Amplification and Illumina MiSeq sequencing of 16S rRNA gene V3-V4

Representative samples for each of the studied areas were prepared individually by combining 0.5 L seawater of appropriate sampling stations and subjected to nucleic acid extraction as described in Supplementary methods. At this step, to prepare representative composite samples for each area, equimolar concentrations of nucleic acids were pooled from all 12 months and composite samples were prepared for area IA (ComI), OA1 (ComO1) and OA2 (ComO2). Thus along with composite samples, a total of 42 nucleic acid samples were selected for amplicon sequencing. These samples were further classified as two broad groups: first group (termed as control) includes n = 16 (13 samples of area IA and 3 samples of area AA), whereas second group (termed as thermal discharge-impacted) includes n = 26 (13 samples each from areas OA1 and OA2).

Amplicons libraries were generated by following the standard Illumina 16S rRNA gene library preparation procedure using universal bacterial primers 341F (5′-CCTACGGGNGGCWGCAG-3′) and 805R (5′-GACTACHVGGGTATCTAATCC-3′)^34^ linked with Illumina adapter and barcode sequence. The primer pair selected here encompasses the V3–V4 hypervariable regions of 16S rRNA gene and has been frequently utilized in several 16S rRNA gene-based diversity studies^33,35^, as it offers a better analysis of bacterial diversity. The detailed protocol on amplicons library preparation has been discussed in our earlier studies^36,37^. The size and quality of prepared libraries were validated by running on Bioanalyzer 2100 (Agilent technologies, California, US) and taken in equal concentrations for paired-end (300 × 2 bp) sequencing on the Illumina MiSeq sequencing platform at Macrogen Inc. (Seoul, Korea). Illumina-generated amplicon reads (iTags) were then processed through bioinformatics analyses.

### Bioinformatics analyses of iTags and statistical analyses

Bioinformatics analyses of our study included quality control and diversity analysis of iTags. Initially, raw Illumina paired-end reads (R1 and R2 fastq files) were quality evaluated in FastQC software^38^. Standard quality control criteria were implemented to filter out low-quality sequences. Briefly, reads shorter than 200 bp in length, adapter sequences and base calls having an average Phred quality score < 30 were discarded using Cutadapt programme^39^. Qualified paired-end reads were assembled and the resulting high-quality full-length reads were analyzed with Quantitative Insights Into Microbial Ecology (QIIME) pipeline version 1.9.0^40^. In QIIME environment, processed sequences were clustered into operational taxonomic units (OTUs) using classical open-reference OTU picking strategy with UCLUST (v1.2.22) algorithm. Representative sequences were chosen for each OTU and were taxonomically assigned at global identity threshold of 97% against Greengenes (13_8_release) reference database^41^. In order to escape from the possible sequencing bias and remove sparse OTUs, we omitted any OTUs comprised of merely single reads (singletons). Here, to achieve identical sequencing depth, sequences from larger samples were randomly sub-sampled to the sequences of smallest individual sample (79, 670 sequences in sample AprO_2_), which enabled comparison among the studied sample groups and offers true biological interpretation^42^. Alpha diversity metrics including number of OTUs, Chao1, Shannon index and Simpson evenness were estimated in QIIME at an even sequencing depth.

Differences in environmental variables across studied areas were determined using one-way analysis of variance (ANOVA) in SPSS statistical software (Chicago, IL, United States). Similarly, alpha diversity indices between sample groups were compared using unpaired *t*-test. Differentially abundant taxonomic groups between the samples of control and thermal discharge-impacted areas were elicited and plotted using STAMP software (version 2.1.3). To compare overall community differences between sample groups, beta diversity based on weighted and unweighted UniFrac^43^ distances was determined on rarified data and visualized in principal coordinate analysis (PCoA). To calculate the *p*-values and test for significant differences between group-specific community compositions, we used analysis of similarity (ANOSIM, 999 permutations) function in QIIME.

### Identification of biomarker bacterioplankton groups

Bacterioplankton community of both groups was further analyzed using linear discriminant analysis (LDA) effect size (LEfSe) algorithm at the online Galaxy interface (http://huttenhower.sph.harvard.edu/galaxy). Default parameters (alpha value of Kruskal–Wallis test was 0.05 and logarithmic LDA threshold score was 2.0) were used and discriminatory bacterial taxa that characterize the bacterioplankton community of seawater with and without thermal-discharge influence are displayed as cladogram and bar graph.

### Molecular ecological network (MEN) construction and characterization

To elucidate the influence of thermal discharge on overall microbial interactions, phylogenetic molecular ecological networks (pMENs) were constructed using molecular ecological network analysis (MENA) pipeline (http://ieg2.ou.edu/MENA) through Random Matrix Theory (RMT)-based approach^44^. As previously suggested^45^, top abundant OTUs (> 0.01%) per sample group were initially log-transformed and then used to obtain a similarity matrix using Pearson correlation. To ensure reliability, OTUs appeared in more than 50% of the total samples were only considered for both groups. The obtained similarity matrix was then threshold scanned through the RMT-based approach to obtain adjacency matrix by applying identical similarity threshold (0.74) for both control and thermal discharge-impacted sample groups. Based on the adjacency matrix, co-occurrence networks were graphed using Gephi software (version 0.9.2). Network topological properties such as module numbers, node numbers, link numbers, R^2^ of power-low, average degree, average clustering coefficient, average path distance and density of networks were compared between both the groups.

Further, topological roles of all nodes were calculated based on within-module connectivity (*Zi*) and among-module connectivity (*Pi*) as described earlier^46^ and displayed as *Z-P* plot. Here, all nodes were categorized into following four categories: (i) peripherals (*Zi* ≤ 2.5, *Pi* ≤ 0.62), these nodes possess only a few links with almost inside their own modules; (ii) connectors (*Zi* ≤ 2.5, *Pi* > 0.62), which are highly linked to several other modules; (iii) module hubs (*Zi* > 2.5, *Pi* ≤ 0.62), which are highly connected to numerous nodes in their own modules; and (iv) network hubs (*Zi* > 2.5, *Pi* > 0.62), which act as both module hubs and connectors.

## Results

### Environmental parameters and seawater quality

During most of the sampling months, the average temperature at thermal discharge-impacted areas (area OA1 and OA2) was higher than control areas (IA and AA), except for those months when the power plant was not in operational status (Supplementary Fig. S2a and Supplementary Table S1). The average temperature ranged between 26 and 32 °C at control areas, whereas, it ranged between 27.6 and 34.5 °C at thermal discharge-impacted areas. The average temperature difference between control and thermal discharge-impacted area OA1 (ΔT1) and area OA2 (ΔT2) ranged between 0 and 6.5 °C (Supplementary Fig. S2b). The content of DO followed similar trends among the studied areas with a concentration range of 4.52 to 7.28 mg O_2_ L^−1^ (Supplementary Fig. S2c). Spatial variations in the concentration of inorganic nutrients such as nitrite, nitrate, SRP and ammonium are shown (Supplementary Fig. S2d–g). As can be seen, no particular pattern of variations in the concentrations of nitrite and phosphorus was observed between the studied areas. However, an upsurge in nitrate and a decline in ammonium concentration were observed throughout the sampling campaign at thermal discharge-impacted areas.

### Overview of iTag sequencing data

A total of 18.288 million paired-end raw reads were generated from analyzed samples (Supplementary Table S2). Employment of quality control criteria resulted in a total of 5.842 million high-quality sequences averaging 139,111 sequences per sample with an average sequence length of 455 ± 11 bp. Bacterial richness (number of OTUs) varied significantly across analyzed samples (Supplementary Fig. S3). Majority of control samples (blue colour curves) had higher richness than thermal discharge-impacted samples (red colour curves). Also, the sequencing effort employed in the present study appeared technically sufficient to capture phylogenetic information, as supported by rarefaction curves that reached saturation stage with the growing sequencing depth.

### Bacterioplankton profile varies between the samples of control and thermal discharge-impacted areas

**Figure 1:**
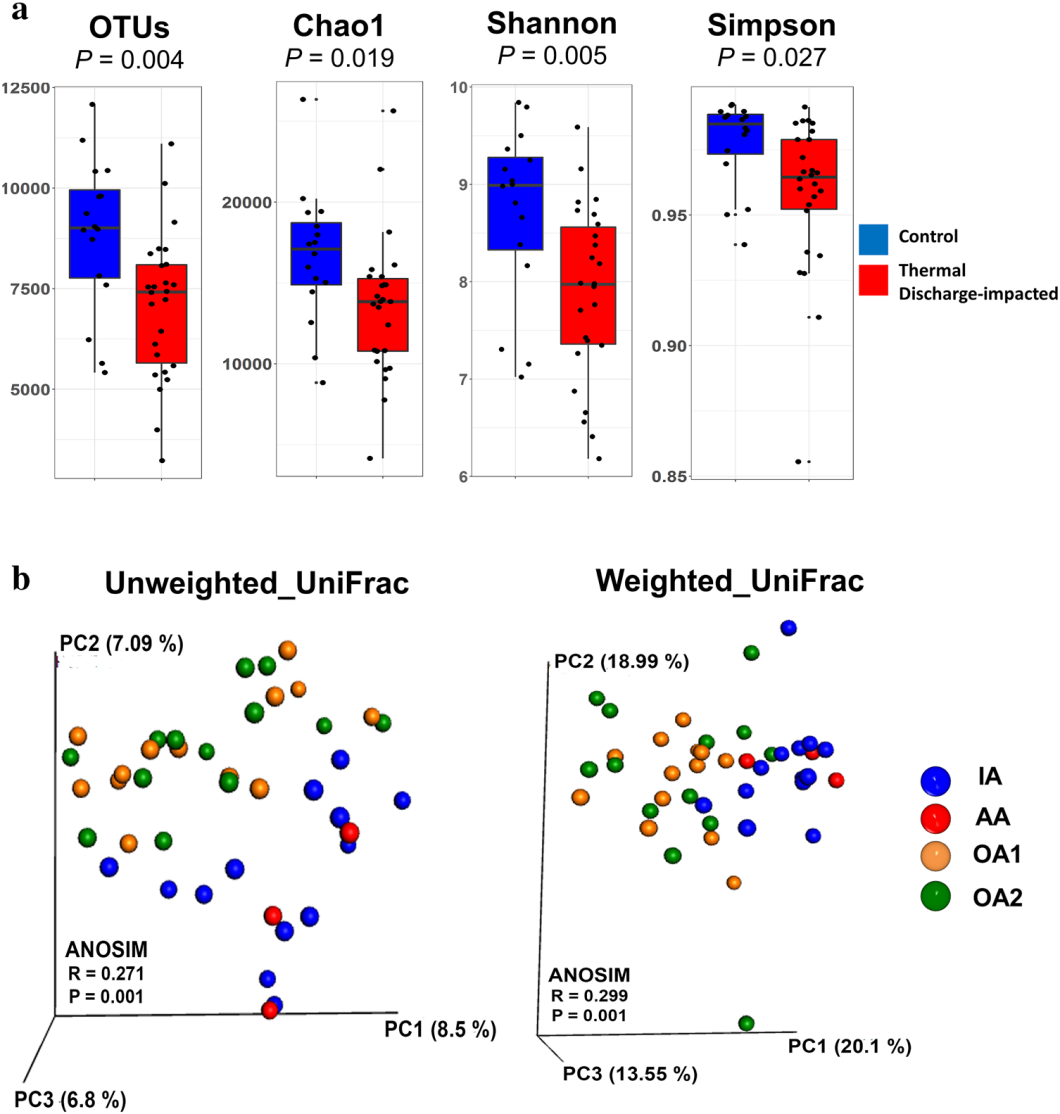
Bacterioplankton richness and community composition variations between control and thermal discharge-impacted samples. (**a**) Box plots displaying the alpha-diversity (OTUs, Chao1, Shannon, and Simpsons) comparison between the control (n = 16) and thermal discharge-impacted (n = 26) groups. The bacterioplankton richness and evenness of control and thermal discharge-impacted samples are clearly distinct (Student’s t-test, *p* < 0.05). (**b**) Principal coordinate analysis (PCoA) plots showing beta diversity variations based on unweighted and weighted UniFrac distance metrics. The bacterioplankton community structure significantly differed between control and thermal discharge-impacted areas as confirmed by ANOSIM (*p* < 0.001).

To evaluate bacterial community variations, we measured alpha diversity (i.e. diversity within sample) and beta diversity (i.e. diversity variations among samples). For alpha diversity variations, bacterial communities of unimpacted control areas and thermal discharge-impacted areas were compared in terms of species richness (number of OTUs and Chao1), diversity (Shannon) and richness as well as evenness (Simpsons). The median values of OTUs (7062) and Chao1 (13,517) in thermal discharge-impacted samples were significantly lower (*p* < 0.005) in comparison to corresponding control samples (OTUs; 8843, Chao1; 16,624) (Fig. 1a). Values of other diversity indices also followed a similar trend, showing a significantly higher bacterial diversity and evenness in samples of control group as estimated by Shannon (*p* < 0.01) and Simpson (*p* < 0.05), respectively.

For beta diversity variation, both groups were compared using phylogenetic distance metrics such as unweighted (qualitative measurement) and weighted (quantitative measurement) UniFrac. Clustering pattern of analyzed samples on PCoA plots indicated that regardless of the sampling months, bacterioplankton community had a strong clustering pattern according to the sampling areas. Therefore, most of the control samples (samples of areas IA and AA) formed a different cluster from the thermal discharge-impacted samples (samples of areas OA1 and OA2) in both unweighted UniFrac-based and weighted UniFrac-based PCoA (Fig. 1b). It is important to emphasize here that the discrimination between both groups is more with weighted UniFrac (20.1% at PC1 and 18.99% at PC2) than with the unweighted UniFrac (8.5% at PC1 and 7.09% at PC2), indicating that the overall community variation is higher based on community structure rather than community membership. Moreover, bacterioplankton communities of control group were more similar to each other than their corresponding heterogeneous communities in thermal discharge-impacted group. Further statistical analysis revealed that the community differences were significant using both unweighted (ANOSIM R value = 0.271, *p* = 0.001) and weighted (ANOSIM R value = 0.299, *p* = 0.001) UniFrac.

### Bacterioplankton community structure differed between control and thermal discharge-impacted samples

**Figure 2:**
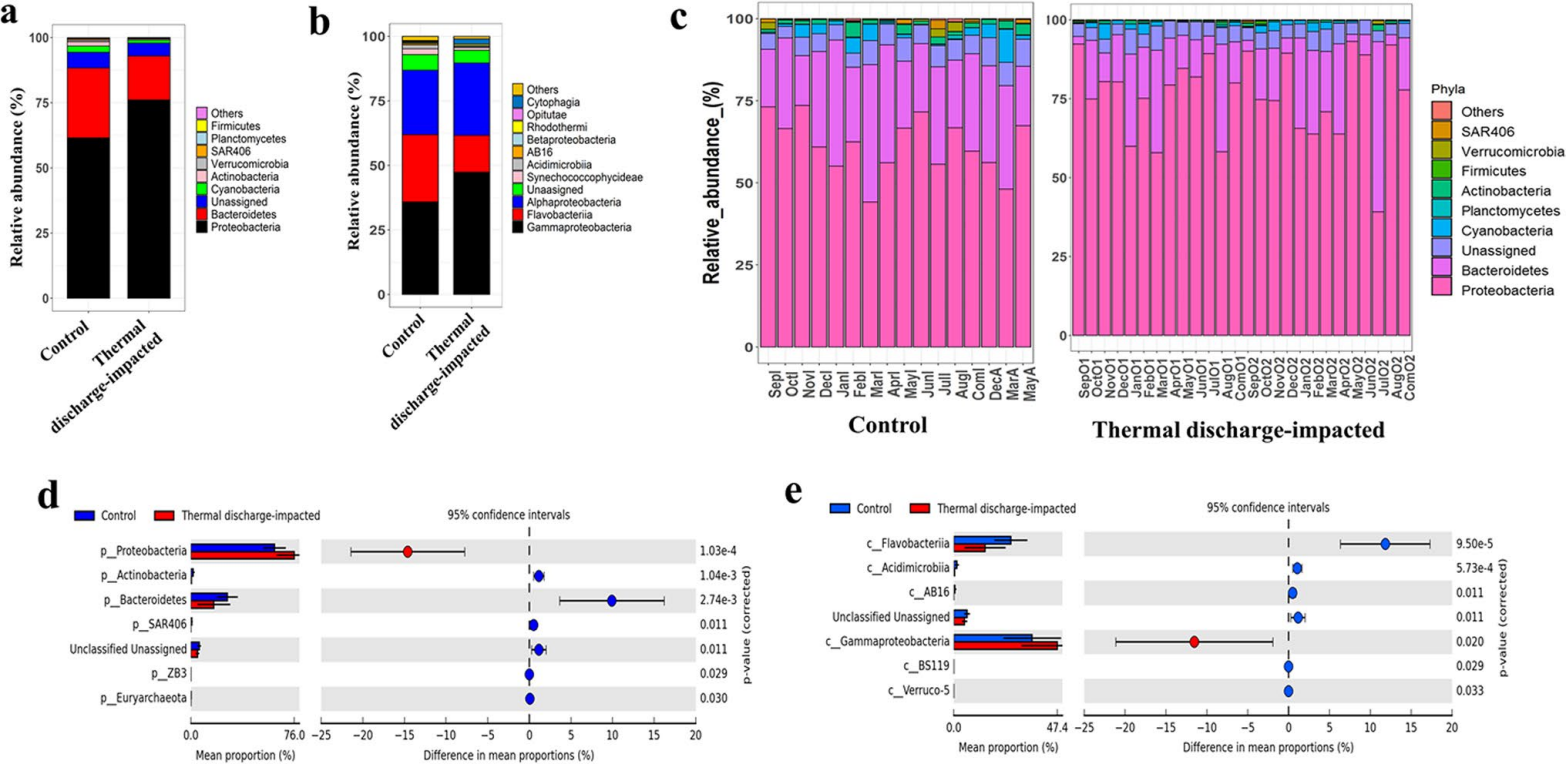
Taxonomic composition of top bacterioplankton groups. (**a**, **b**) Stacked bar plots depicting the average relative abundance of dominant bacterioplankton taxa at (**a**) phylum and (**b**) class levels in control (n = 16) and thermal discharge-impacted (n = 26) sample groups. (**c**) Month-wise relative abundance of bacterioplankton community. (**d**, **e**) Extended error bars displaying the variation in proportion of sequences of significantly differed bacterioplankton groups between control and thermal discharge-impacted areas at (**d**) phylum and (**e**) class level. Only those taxonomic groups are represented whose abundances constituted ≥ 0.5% of the total community.

The relative abundances of top dominant bacterioplankton taxa, each constituted for the relative abundance of ≥ 0.5% of total community is represented at phylum and class level (Fig. 2a,b). As can be visualized in Fig. 2a, though bacterial communities of both groups were dominated by phyla *Proteobacteria*, *Bacteroidetes*, *Cyanobacteria*, *Planctomycetes*, *Actinobacteria*, *Firmicutes*, *Verrucomicrobia* and *SAR406*, their mean relative proportions varied considerably. Throughout the sampling campaign, phylum *Proteobacteria* was found to be high in thermal discharge-impacted samples (range = 39.16%–93.12%; mean abundance = 76.05%) when compared to control samples (range = 44.15%–66.69%; mean abundance = 61.50%) (Fig. 2a,c). In contrast to *Proteobacteria*, a consistent opposite trend (proportionally less at thermal discharge-impacted areas and high at control areas) of *Bacteroidetes* was observed. The group-specific differences were then compared using extended error bars option in STAMP software. As expected, a significantly (*p* < 0.05) higher mean proportion of phylum *Proteobacteria* in thermal discharge-impacted samples and a higher proportion of *Bacteroidetes* in control samples were observed (Fig. 2d). Further insight at the class level indicated that an increase in *Gammaproteobacteria* abundance at thermal discharge-impacted areas and a decrease in *Flavobacteriia* were primarily responsible for differences that occurred between the control and thermal discharge-impacted groups (Fig. 2e).

To decipher month-wise differences in bacterioplankton community, heatmaps were constructed at phylum level (Supplementary Fig. S4). Of the studied twelve months, most of the months showed differential clustering of control samples from thermal discharge-impacted. For instance, area IA clustered separately from the areas OA1 and OA2 during most of the sampling occasions. It was also interesting to note that the samples of area AA clustered separately from the areas OA1 and OA2 along with area IA, indicating that the bacterial communities of ambient and intake areas were compositionally similar.

**Figure 3:**
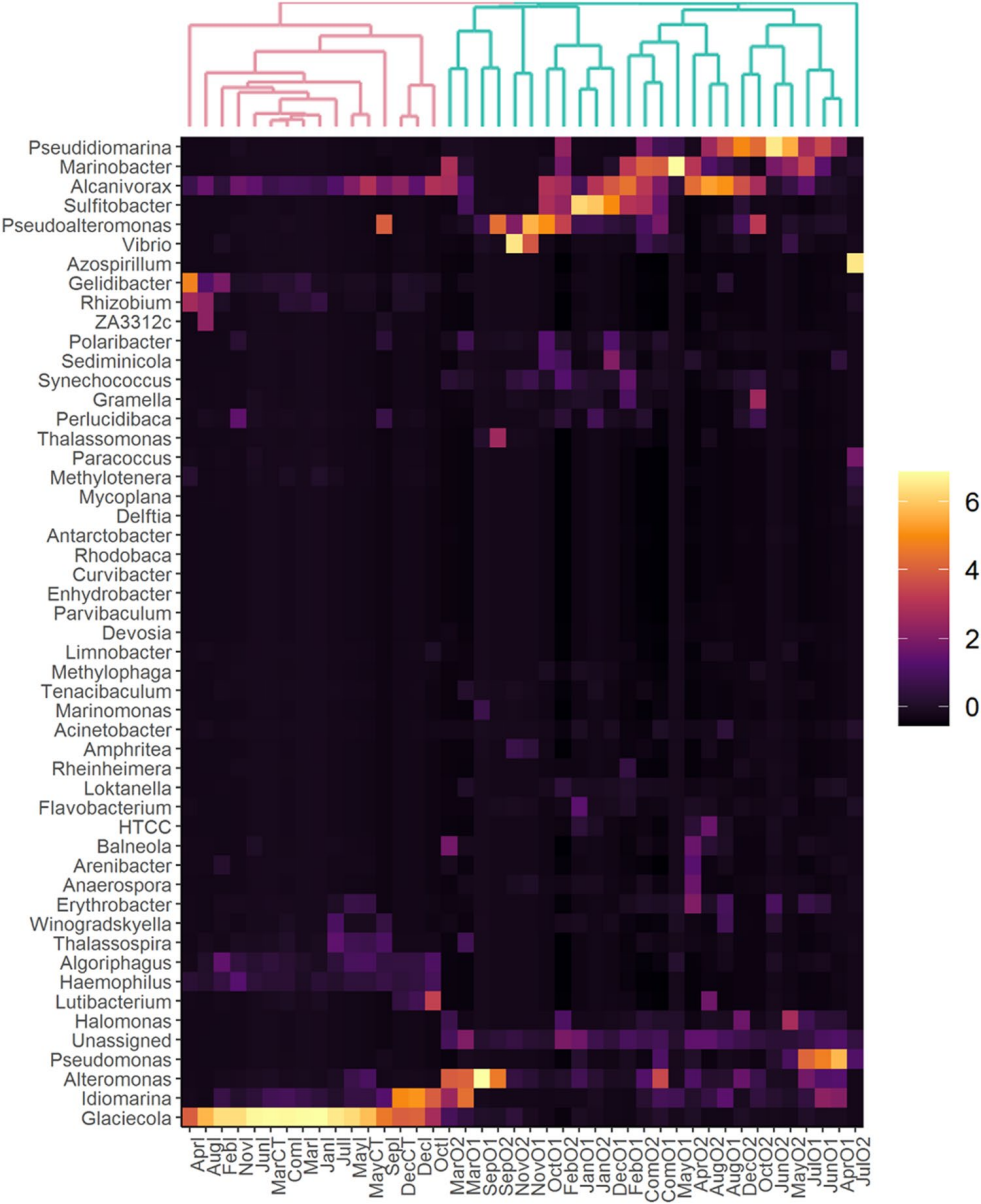
Bacterial community comparison at genus level. Hierarchically clustered heatmap representing the relative abundances of most abundant bacterial genera in seawater of control and thermal discharge-impacted areas. The colour intensity in each panel signifies the relative proportion (%) of respective bacterial genera in a sample. The samples of control groups (shown as pink colour cladogram) clustered separately from those of thermal discharge-impacted groups (shown as green colour cladogram).

To study the influence of thermal discharge at lower taxonomic rank, iTag sequences were also analyzed at genus level (Fig. 3). As can be inferred by clustering pattern in heatmap, the bacterioplankton communities of control and thermal discharge-impacted areas were characterized by distinct bacterial genera. For instance, the average relative abundance of several genera such as *Alcanivorax* (0.07% versus 5.12%), *Alteromonas* (0.00% vs 5.52%), *Enhydrobacter* (13.38% vs 0.00%), *Glaciecola* (11.95% vs 0.86%), *Marinobacter* (1.17% vs 5.09%), *Parvibaculum* (4.38% vs 0.01%), *Pseudidiomarina* (2.75% vs 5.45%), *Pseudoalteromonas* (0.00% vs 4.89%), *Pseudomonas* (0.00% vs 2.20%), *Sulfitobacter* (1.00% vs 3.50%), *Acinetobacter* (0.06% vs 1.16%) and *Vibrio* (0.50% vs 2.87%) varied between control and thermal discharge-impacted groups. To tease out which bacterial genera varied significantly, data was statistically evaluated using STAMP software. Most importantly, a total of 10 bacterial genera (*Glaciecola*, *Algoriphagus*, *Candidatus Portiera*, *R562*, *Sphingomonas*, *Lutibacterium*, *Owenweeksia*, *Ralstonia*, *Cellulophaga*, and *Rhodobaca*) in control and 14 genera (*Synechococcus*, *Acinetobacter*, *Pseudidiomarina*, *Marinobacter*, *Halomonas*, *Sulfitobacter*, *Alteromonas*, *Pseudoalteromonas*, *Flavobacterium*, *Perlucidibaca*, *Anaerospora*, *Sediminicola*, *Pseudomonas*, and *Gramella*) in thermal discharge-impacted areas were identified, whose abundances were found to be significantly overrepresented (*p* < 0.05) (Supplementary Fig. S5).

### Specific bacterioplankton taxa are associated with control and thermal discharge-impacted sample groups

**Figure 4:**
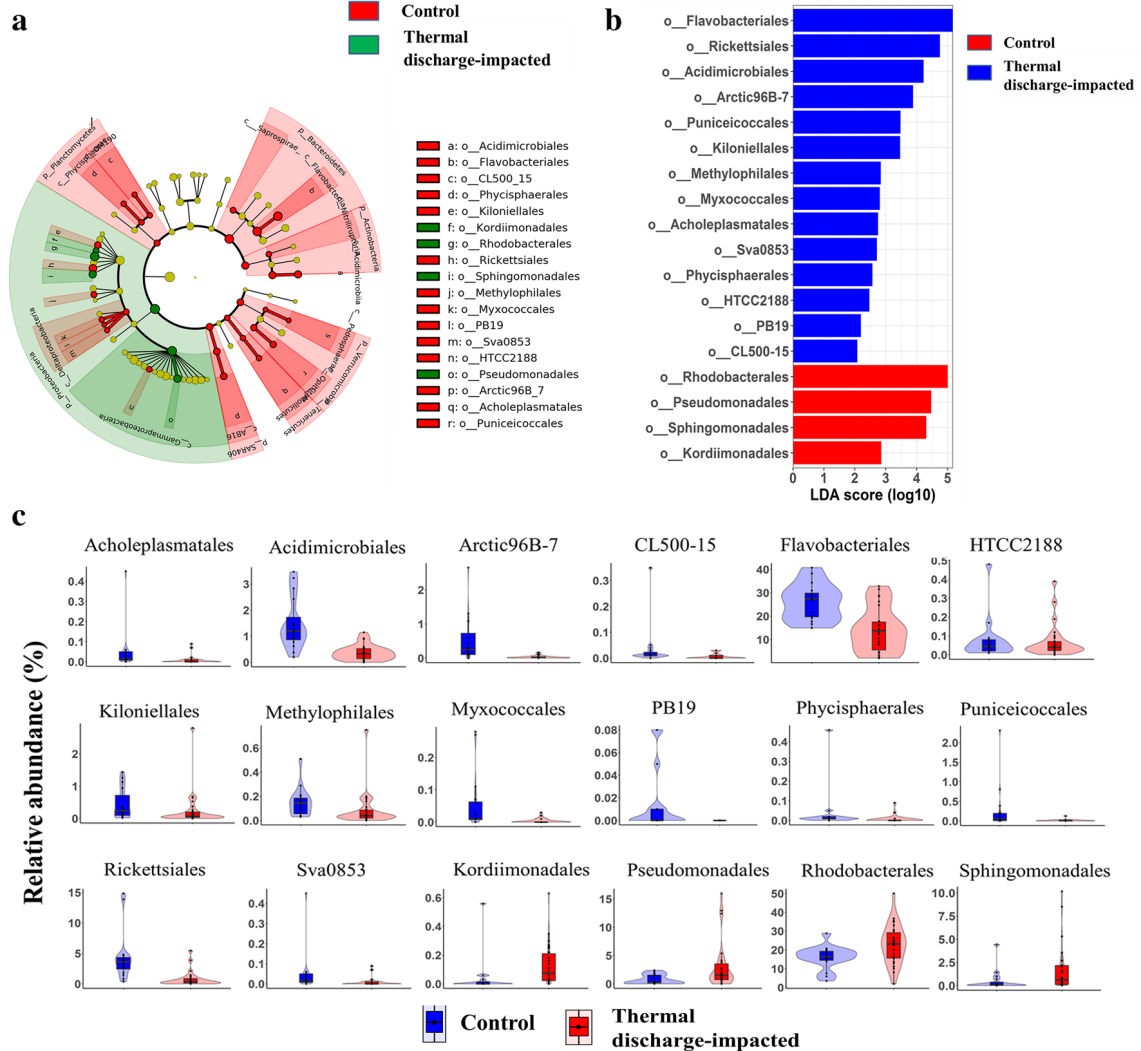
The most differentially abundant bacterioplankton groups. (**a**) Taxonomic cladogram based on linear discriminant analysis (LDA) effect size (LEfSe) illustrating the bacterioplankton communities with significantly differential abundance. Red highlighted region in cladogram indicates bacterioplankton taxa enriched in control samples, while region with green highlights are those enriched in thermal discharge-impacted samples. (**b**) Histogram of differentially abundant bacterioplankton taxa identified with LDA score ≥ 2. (**c**) The violin and boxplots showing relative proportion of differentially abundant taxa identified through LEfSe analysis.

Bacterioplankton community composition was then analyzed (at order level) using LEfSe programme to identify bacterial groups that consistently and significantly varied between control and thermal discharge-impacted samples (Fig. 4). LEfSe-based cladogram indicated a total of 14 and 4 taxonomic biomarkers in control and thermal discharge-impacted areas, respectively. Of these, the relative abundance of *Kordiimonadales*, *Rhodobacterales*, *Sphingomonadales* and *Pseudomonadales* were more enriched (LDA score > 2) in thermal discharge-impacted samples (Fig. 4a,b). Conversely, microbial community of control samples was characterized by significant preponderance (LDA score > 2) of *Acidimicrobiales*, *Flavobacteriales*, *CL500_15*, *Phycisphaerales*, *Arctic96B-7*, *Kiloniellales*, *Rickettsiales*, *Methylophilales*, *Myxococcales*, *Acholeplasmatales*, *Sva0853*, *HTCC2188*, *PB19* and *Puniceicoccales*. The proportions of these differentially abundant taxonomic markers were then visualized as violin and box plots (Fig. 4c).

### Influence of thermal discharge-created warming on co-occurrence ecological network

**Figure 5:**
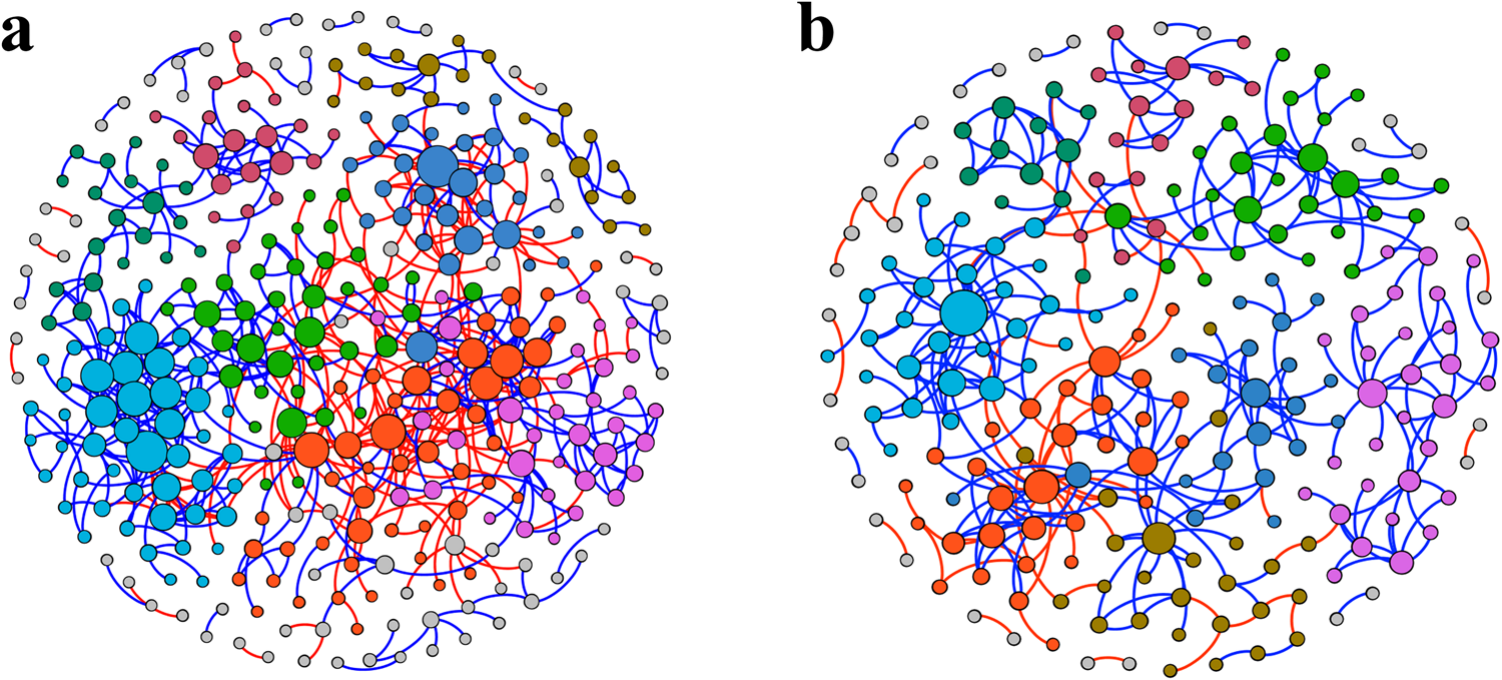
The co-occurrence ecological networks of marine bacterioplankton communities. (**a**, **b**) Phylogenetic molecular ecological networks (pMENs) depicting the bacterioplankton community interactions differences between control (**a**) and thermal discharge-impacted (**b**) areas. The node (OTUs) colour signifies the modularity class of the OTUs, and the node size is proportional to their connectivity values (node degree). Blue and red bridging edges represent positive and negative links, respectively among the nodes.

To elucidate the influence of thermal discharge on bacterioplankton community interactions, co-occurrence networks were constructed (Fig. 5a,b). The topological properties of constructed networks are represented (Supplementary Table S3). Although same threshold values were used for construction, network of control group was found to be more larger, having a higher number of nodes (330), higher number of edges (620), shorter average path distance (6.26) and higher density (0.011) compared to the smaller networks of thermal discharge-impacted areas (228 nodes, 340 edges, 6.47 average path distance and 0.010 density). Also, the number of modules (22) in thermal discharge-impacted group was reduced than control (31) and was less interactive. On the contrary, all the nodes in the control network were connected to several modules. Overall, the thermal discharge-impacted network had reduced complexity and connectivity than the control network, indicating higher bacterioplankton interactions in the control group as compared to the thermal discharge-impacted.

**Figure 6:**
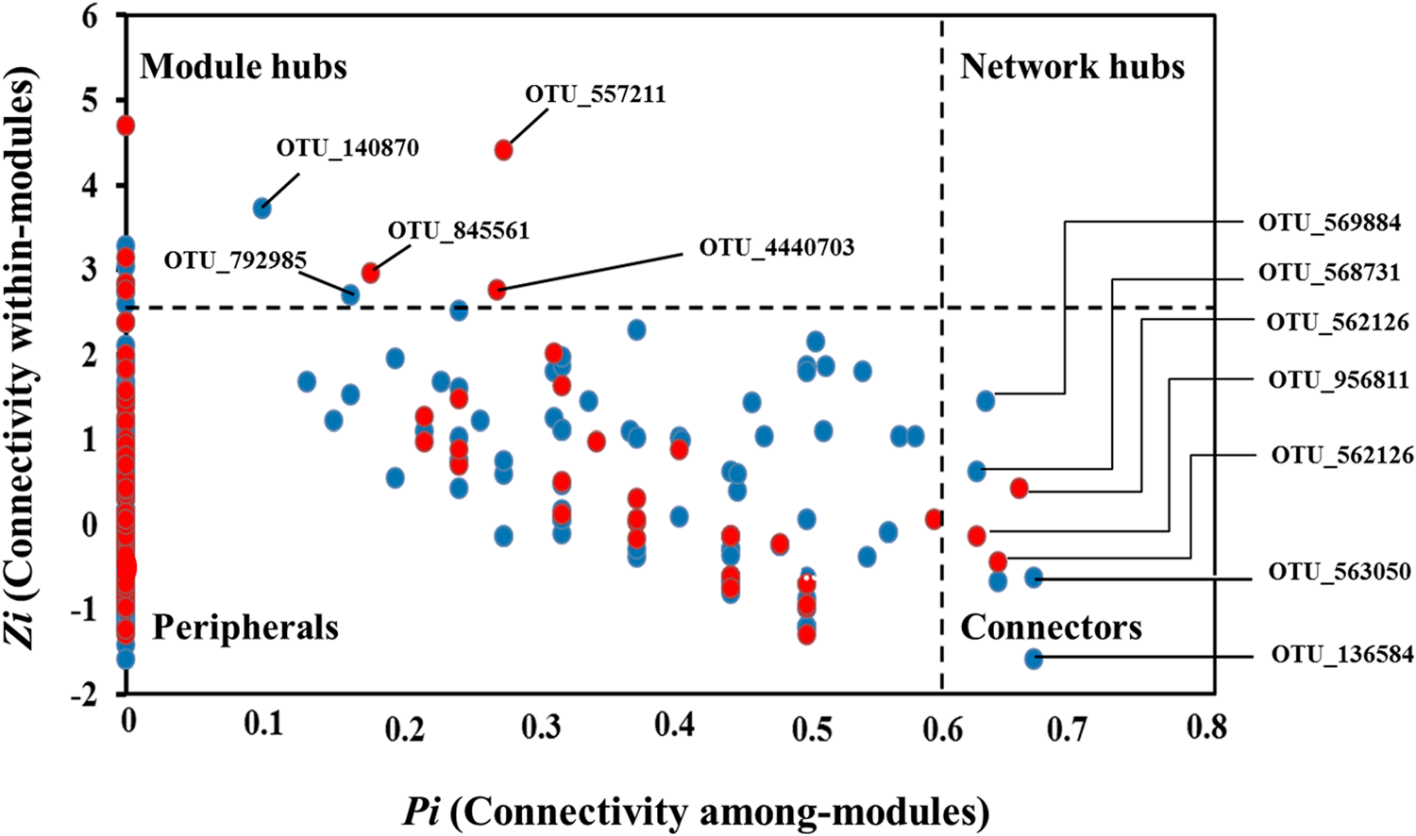
Classification of nodes based on their topological roles. *Z-P* plot showing distribution of nodes based on their within-module connectivity (*Zi*) and among-module connectivity (*Pi*) values to identify putative keystone taxa in co-occurrence networks of control and thermal discharge-impacted samples. Each dot represents a node belonging to control (blue colour) and thermal discharge-impacted (red colour) area.

The topological roles of all nodes were then determined and visualized on a *Z–P* plot (Fig. 6). Majority of nodes (> 98%) present in both groups were identified as peripherals, with most of their link inside their own modules. In the case of thermal discharge-impacted group, even 83% of the peripheral nodes are not at all connected outside their own module (*Pi* = 0). Following peripherals, a total of 4 nodes (OTU136584, OTU569884, OTU563050 of *Flavobacteriia* and OTU568731 of *Alphaproteobacteria*) were in control group whereas, 3 nodes (OTU589792, OTU956811 of *Gammaproteobacteria* and OTU562126 of *Alphaproteobacteria*) in thermal discharge-impacted group served as connectors (Fig. 6 and Table S4). In addition, 2 nodes (OTU140817 and OTU792985 of *Gammaproteobacteria*) in control group and 3 nodes (OTU4440703 of *Gammaproteobacteria*, OTU557211 of *Cyanobacteria* and OTU845561 of *Alphaproteobacteria*) in thermal discharge-impacted group served as module hubs. Nodes falling under network hubs were not identified in both of the studied sample groups.

## Discussion

The ecological consequences of elevated temperature have been debated for a long time and more in-depth studies are required to decipher its impact on marine ecosystem. A large volume of thermal effluent discharged from power plants create temperature gradient in the receiving water body, which provides an ideal model to delineate probable response of bacterioplankton community to climatic changes especially, global warming^20,31^.

Therefore, in the present study, we profiled bacterioplankton community associated with control and thermal discharge-impacted seawater to gain better understanding on how marine bacterioplankton community responds to elevated temperature. Based on nearly six million high-quality iTags, we demonstrated that bacterioplankton community in control seawater is significantly varied from seawater impacted with thermal discharge. Bacterioplankton community of thermal discharge-impacted seawater is characterized by reduced diversity and richness. These results suggest that there is a loss of bacterial communities in thermal discharge-impacted areas, which corroborates with previous studies and theoretical prediction showing decreasing bacterial richness with increasing temperature^47,48^. Here, one reason for the observed difference in bacterioplankton richness might be due to their differential response against elevated temperature. Bacterioplankton taxa in a given perturbation are either sensitive or resistant. In case if the communities are sensitive, then a particular species will decrease/increase or replace one another^49^, which ultimately influences bacterioplankton richness as well as evenness and thereby ecosystem services^50,51^.

Furthermore, unweighted and weighted UniFrac-based PCoA showed an increase in beta diversity pattern and therefore a clear distinction in bacterioplankton community composition of control and thermal discharge-impacted areas was observed. These results suggest that elevated seawater temperature causes substantial shift in marine bacterioplankton community. There is mounting evidence that stressors including overfishing, pollution and elevated temperature increase microbial beta diversity in marine environment^52^. Similar results were also previously observed by other studies where elevated temperature-induced significant alteration in bacterial community of freshwater^53^ and seawater^15^. Nevertheless, few studies have demonstrated that synergistic influence of two disturbing factors is greater than their individual influences. For example, Ren et al.^16^ reported that elevated temperature alone had insignificant effects, whereas elevated temperature in combination with nutrient enrichment causes a significant shift in freshwater bacterioplankton community. Similarly, a mesocosms study by Lindh et al.^14^ indicated that the elevated temperature combined with acidification imposes significant impact on marine bacterioplankton community.

Besides reduced richness and varied composition of overall bacterioplankton community that we detected in thermal discharge-impacted areas, a contrasting pattern in the relative proportion of two marine bacterial phyla such as *Proteobacteria* and *Bacteroidetes* was observed throughout the sampling campaign. For instance, a significantly higher proportion of *Proteobacteria* and lower of *Bacteroidetes* was observed at thermal discharge-impacted areas. Members of phylum *Proteobacteria* and *Bacteroidetes* are considered as most abundant bacterial groups in the marine environment^54^. The increase in relative abundance of phylum *Proteobacteria* especially, class *Gammaproteobacteria* at thermal discharge-impacted areas might be attributed to the copiotrophic lifestyle of this taxa because of which it responds rapidly to environmental disturbances and thus become dominant member in a community^55^. Therefore, elevated seawater temperature has been frequently shown to increase *Gammaproteobacteria* abundance in various in situ studies conducted in Baltic Sea^56,57^, Mediterranean Sea^58^, and North Sea^59^. Contrary to *Proteobacteria*, phylum *Bacteroidetes* was negatively influenced by elevated temperature in our study. Generally, proportion of phylum *Bacteroidetes* ranges between 10 and 30% of total bacterial communities in marine coastal areas^60^, and nearly same abundance range (15–41%) was observed throughout the sampling at control areas in our study. However, a decreased proportion (> 10%) during most of the sampling months at thermal discharge-impacted areas was observed. A negative correlation between elevated temperature and *Bacteroidetes* abundance has also been previously reported in a freshwater mesocosms study^16^.

Identification of taxonomic biomarkers for various anthropogenic factors is a critical task in ecology, especially in case of microbes owing to their extreme complexity and high diversity^45^. In this backdrop, we tried to identify specific bacterial biomarker taxa for thermal discharge-impacted samples using LEfSe. This analysis identified three orders (*Kordiimonadales, Rhodobacterales*, *Sphingomonadales*) of *Alphaproteobacteria* and one order (*Pseudomonadales*) of *Gammaproteobacteria*, whose abundance was significantly enhanced under elevated temperature. Here, it is important to note that a few bacterial genera belonging to these taxonomic biomarkers such as *Pseudomonas* and *Acinetobacter* (members of *Pseudomonadales*) and *Sulfitobacter* (member of *Rhodobacterales*) are generally considered as potential opportunistic pathogens. The increasing seawater temperature and other stressors (e.g. pollutants and overfishing) have often been shown to favour the proliferation of opportunistic pathogens^52^. For example, few members of *Rhodobacterales* are fast growing opportunistic pathogens that have been shown to bloom under stress condition when there is an open niche space^61^. Moreover, under warming condition, members of *Rhodobacterales* exert higher activation energy which helps in out-competing other bacterial taxa in community^62^. Similar to *Rhodobacterales*, members of order *Sphingomonadales* have been previously linked with elevated temperature^63,64^. We also observed an exclusive dominance of a few genera (e.g. *Vibrio*) belonging to order *Vibrionales* at thermal discharge-impacted areas, which is unsurprising as members of *Vibrionales* have been frequently reported to increase under thermal stress conditions^65,66^. A notable study by Vezzulli et al.^19^, who investigated plankton-associated microbiota in southern North Sea divulged that ocean warming is favouring the dominance of marine pathogenic bacteria especially, members of *Vibrio* genus including *V. cholera*. Similarly, increasing evidence has also been gathered associating geographical and seasonal expansion of seafood-borne diseases caused by *V. parahaemolyticus* and *V. vulnificus* with climatic anomalies^67^. In addition to potential pathogenic genera, the effect of elevated temperature on the blooming of certain other marine bacterial genera such as *Marinobacter*, *Pseudoalteromonas*, *Alteromonas*, *Pseudidiomarina*, and *Halomonas* was also observed.

Majority of the previous studies that dealt with assessing the influence of elevated temperature on marine bacterioplankton have been focused on their richness and community composition^20,21^, but not on their interactions. However, community interactions could be an important component than species richness and composition, especially in complex ecosystems where it directly influences the ecosystem stability and functioning^44,68^. Considering this, co-occurrence ecological networks were constructed. A considerable alteration in the co-occurrence network of thermal discharge-impacted areas was observed in terms of network structure, network organization, network composition, topological roles of individual nodes, and network hubs. For example, number of modules, nodes, connectivity/density, clustering coefficient and links were lower in thermal discharge-impacted group. These results reveal that the elevated temperature causes a profound influence on microbial interactions pattern along with their richness and community composition. A plausible explanation for alteration in microbial co-occurrence network at thermal discharge-impacted areas may be due to the reduced bacterioplankton richness and varied community structure. A number of previous studies have suggested that high diversity is one of the mechanisms that bacterioplankton communities attain to resist against environmental disturbances, a hypothesis termed as the “insurance effect”^69,70^.

In general, the complexity of ecological network indicates the stability of microbial community structure^45,71^. Thus, stress caused by elevated temperature might cause direct influence on bacterioplankton community stability. Though a dearth of information is available on interactions among marine bacterial assemblages under elevated temperature conditions, several reports describing the potential impact of other environmental anomalies such as ocean acidification (reduction in seawater pH) are available. For example, alterations in microbial ecological networks under elevated CO_2_ condition has been observed by Xia et al.^72^. Nevertheless, this is not always the case, as few studies have also found that microbial intra-species interactions among marine microorganisms are either less affected or unaffected in climatic changes such as elevated partial pressure of CO_2_ (*p*CO2)^73^.

Identifying keystone nodes (hubs) in an ecological network is an important aspect as it provides information on most important members of the entire community. From an ecological viewpoint, peripheral nodes are considered as specialist, connectors and module hubs as generalist and network hubs as super-generalist^68^. Specialist nodes generally link to only a few nodes whereas, generalist nodes bridge to several nodes within their own module as well as among other modules^44^. Therefore, generalist nodes are considered as key members in a community. In our study, nodes identified as generalists in control group were mostly belonging to class *Flavobacteriia* and *Alphaproteobacteria* whereas, *Gammaproteobacteria* and *Alphaproteobacteria* in thermal discharge-impacted group. These data indicated that elevated temperature greatly influences the topological role of individual nodes and also alters the key microbial population in marine environment.

Overall, the present investigation demonstrated an important aspect of marine ecology that elevated seawater temperature has profound influence on bacterioplankton richness, community composition and co-occurrence interaction pattern. Moreover, our results demonstrated that bacterial assemblages are suitable indicators for both biomonitoring of anthropogenic perturbations and to determine the ecological health of marine environment. It would be interesting to disentangle the influence of elevated temperature-induced reduction in richness and varied community composition on overall ecosystem functioning and as such should be deliberated in future research.

## Supplementary Information

Supplementary Information.

## Data Availability

Paired-end Illumina reads supporting the findings of the current study are available in Sequence Read Archive (SRA) of NCBI under accession codes SRR13787186-SRR13787227 and BioProject code PRJNA704768.
