# Supplementary material for: Thermal discharge-induced seawater warming alters richness, community composition and interactions of bacterioplankton assemblages in a coastal ecosystem
Source: Sci Rep. 2021 Aug 30;11:17341. doi: 10.1038/s41598-021-96969-2 (PMC8405676; doi:10.1038/s41598-021-96969-2)

Supplemental Material for the manuscript entitled "Thermal discharge-induced seawater warming alters richness, community composition and interactions of bacterioplankton assemblages in a coastal ecosystem"

Meora Rajeev[a], T J Sushmitha[a], Chairmandurai Aravindraja[b], Subba Rao Toleti[c], Shunmugiah Karutha Pandian[a, *]

[a] Department of Biotechnology, Alagappa University, Science Campus, Karaikudi- 630 003, Tamil Nadu, India.

[b] Department of Periodontology, College of Dentistry, University of Florida, Gainesville, FL, USA.

[c] Water and Steam Chemistry Division, Bhabha Atomic Research Centre Facilities, Kalpakkam 603 102, Tamil Nadu, India.

***Corresponding Author's Address:**

Shunmugiah Karutha Pandian

Senior Professor and Head, Department of Biotechnology

Science Campus, Alagappa University

Karaikudi – 630 003, Tamil Nadu, India

Phone (O) + 91 4565 225215

E-mail : sk_pandian@alagappauniversity.ac.in

Supplementary Methods

Physicochemical characteristics and nutrients analyses

Sensitive environmental variable such as temperature was measured on-site with standard mercury thermometer. Dissolved oxygen (DO) content of individual station was immediately fixed as precipitate in 300 mL capacity BOD bottle and determined using modified Winkler titration [Carpenter, 1965]. Concentration of inorganic nutrients such as soluble reactive phosphorus (SRP) and ammonium (NH_4^+-N) were spectrophotometrically analyzed by standard methods [Parsons et al., 1992]. The sequential estimation of nitrite (NO_2-N) and nitrate (NO_3-N) followed the previously suggested protocol [García et al., 2014].

Nucleic acid extraction

Representative seawater samples for each areas were initially coarse filtered through 11 μm Whatman filter papers to remove the debris/solid particles and microbial biomass was subsequently concentrated on 0.22 μm porosity, polycarbonate membrane filters (Millipore, Massachusetts, USA) using low-vacuum peristaltic pump. Filter membranes were frozen at -80°C until further process. Total bacterial community DNA was extracted using PowerWater DNA Isolation Kit (MoBio Laboratories Inc., USA) following instructions from the manufacturer's protocol. The extracted DNA was evaluated on agarose gel (1% agarose in 1 × Tris-acetate-EDTA buffer) for quality and quantified with picogreen (Life Technologies, USA) using Victor 3 fluorometry (PerkinElmer, Inc. USA).

References

1. Carpenter JH. The Chesapeake Bay Institute technique for the Winkler dissolved oxygen method. Limnol Oceanogr. 1965;10:141-3.
2. Parsons TR, Maita Y, Lalli CM. A manual of chemical and biological methods for seawater analysis. Pergamon Press, New York, 1992.
3. García-Robledo E, Corzo A, Papaspyrou S. A fast and direct spectrophotometric method for the sequential determination of nitrate and nitrite at low concentrations in small volumes. Mar Chem. 2014;162:30-6.

Supplementary Tables

Supplementary Table S1. Time period of seawater sampling and power plant status.

**Sampling date**	**Power generation (MWe)**	
	Unit - I	Unit - I
September 23, 2016	Shut down	Shut down
October 26, 2016	1000	380
November 26, 2016	860	540
December 23, 2016	860	680
January 25, 2017	820	1000
February 28, 2017	Shut down	470
March 31, 2017	Shut down	850
April 21, 2017	800	920
May 27, 2017	850	940
June 20, 2017	Shut down	1000
July 11, 2017	Shut down	1000
August 23, 2017	Shut down	Shut down

Supplementary Table S2. Overview of Illumina generated reads (iTags) before and after applying pre-processing and filtering criteria.

Sampling Groups	Sampling areas	Sample ID	Sampling month	Number of raw reads (Paired-end)	Number of high-quality reads	Number of OTUs
Control (n= 16)	IA	SepI	September -2016	408678	113781	5416
		OctI	October -2016	389538	119716	5642
		NovI	November -2016	515444	167554	8980
		DecI	December -2016	443694	155382	8956
		JanI	January -2017	425392	89979	6232
		FebI	February -2017	515596	158938	9367
		MarI	March -2017	452900	141847	7822
		AprI	April -2017	397596	129090	9804
		MayI	May -2017	451092	144526	10416
		JunI	June -2017	434318	132168	7594
		JulI	July -2017	454874	127313	9044
		AugI	August -2017	497830	162533	8724
		ComI	Composite	578134	197763	12081
	AA	DecA	December -2016	263894	105262	11190
		MarA	March -2017	454986	146368	9785
		MayA	May -2017	226152	91313	10436
Thermal discharge-impacted (n = 26)	OA1	SepO1	September -2016	424382	143072	5356
		OctO1	October -2016	324416	116655	5425
		NovO1	November -2016	380760	115768	7642
		DecO1	December -2016	471008	155354	3989
		JanO1	January -2017	368364	99673	7597
		FebO1	February -2017	415972	153572	7541
		MarO1	March -2017	566830	181762	7227
		AprO1	April -2017	424024	124502	5854
		MayO1	May -2017	419616	115437	6441
		JunO1	June -2017	421468	142080	7546
		JulO1	July -2017	424024	143926	7432
		AugO1	August -2017	452490	127870	9152

		ComO1	Composite	527220	167862	11103
	OA2	SepO2	September -2016	514858	180573	6122
		OctO2	October -2016	370850	134461	8477
		NovO2	November -2016	458994	157820	7406
		DecO2	December -2016	406762	99750	7117
		JanO2	January -2017	486656	182611	4997
		FebO2	February -2017	440934	155668	8371
		MarO2	March -2017	499118	154578	8073
		AprO2	April -2017	379884	79670	8102
		MayO2	May -2017	447020	145477	8494
		JunO2	June -2017	425978	154290	5237
		JulO2	July -2017	515126	176343	3223
		AugO2	August -2017	427390	146437	5586
		ComO2	Composite	384632	103418	10113

Supplementary Table S3. Major topological properties of phylogenetic molecular ecological networks (pMENs) of microbial communities associated with control and thermal discharge-impacted groups.

Sample groups	Topological properties							
	Total nodes	Total links	R^2 of power-law	Average degree (avgK)	Average clustering coefficient (avgCC)	Average path distance (GD)	Density	No. of Modules
**Control**	330	620	0.952	3.758	0.185	6.258	0.011	31
**Thermal discharge-impacted**	228	340	0.911	2.982	0.228	6.479	0.010	22

Supplementary Table S4. Information of the module and connectors hubs identified for control and thermal discharge-impacted groups.

Sample groups	Hubs	*Z*i	*P*i	OTU_ID	Taxonomic allocation
Control	Module	2.71	0.16	792985	p__Proteobacteria; c__Gammaproteobacteria; o__Oceanospirillales; f__Halomonadaceae; g__Candidatus Portiera
		3.71	0.09	140870	p__Proteobacteria; c__Gammaproteobacteria; o__Vibrionales; f__Pseudoalteromonadaceae; g__Pseudoalteromonas;
	Connectors	-1.58	0.66	136584	p__Bacteroidetes; c__Flavobacteriia; o__Flavobacteriales; f__Flavobacteriaceae
		-0.61	0.66	563050	p__Bacteroidetes; c__Flavobacteriia; o__Flavobacteriales; f__NS9
		0.63	0.62	568731	p__Proteobacteria; c__Alphaproteobacteria; o__Rhodospirillales; f__Rhodospirillaceae;
		1.45	0.63	569884	p__Bacteroidetes; c__Flavobacteriia; o__Flavobacteriales;
Thermal discharge-impacted	Module	2.76	0.27	4440703	p__Proteobacteria; c__Gammaproteobacteria; o__Alteromonadales; f__HTCC2188; g__HTCC
		2.95	0.18	845561	p__Proteobacteria; c__Alphaproteobacteria; o__Rhodobacterales; f__Rhodobacteraceae;
		4.41	0.27	557211	p__Cyanobacteria; c__Synechococcophycideae; o__Synechococcales; f__Synechococcaceae; g__Synechococcus
	Connectors	0.43	0.65	562126	p__Proteobacteria; c__Alphaproteobacteria; o__Rhodobacterales; f__Rhodobacteraceae
		-0.44	0.64	956811	p__Proteobacteria;c__Gammaproteobacteria; o__Alteromonadales; f__Idiomarinaceae; g__Idiomarina
		-0.13	0.625	589792	p__Proteobacteria; c__Gammaproteobacteria; o__Pseudomonadales; f__Moraxellaceae; g__Psychrobacter

Supplementary Figures

Supplementary Figure S1. Sampling sites map showing twelve sampling stations (St.1-St.12) of surface seawater collection in the vicinity of a power plant located in the southern coastal region of India. The colours indicate the sampling areas viz. (i) red = outfall area 1 (OA1) that covers St.1 to St.3, (ii) black = outfall area 2 (OA2) covering St.4-St.6, (iii) blue = intake area (IA) covering St.7-St.9 and, (iv) green = ambient area (AA) covering St.10-St.12. The map and sampling sites represented here were created through tools in Google Earth Pro software (v7.3.3.7699) (https://www.neowin.net/news/google-earth-pro-7337699) and was further improved in Adobe Photoshop CC 14.2.

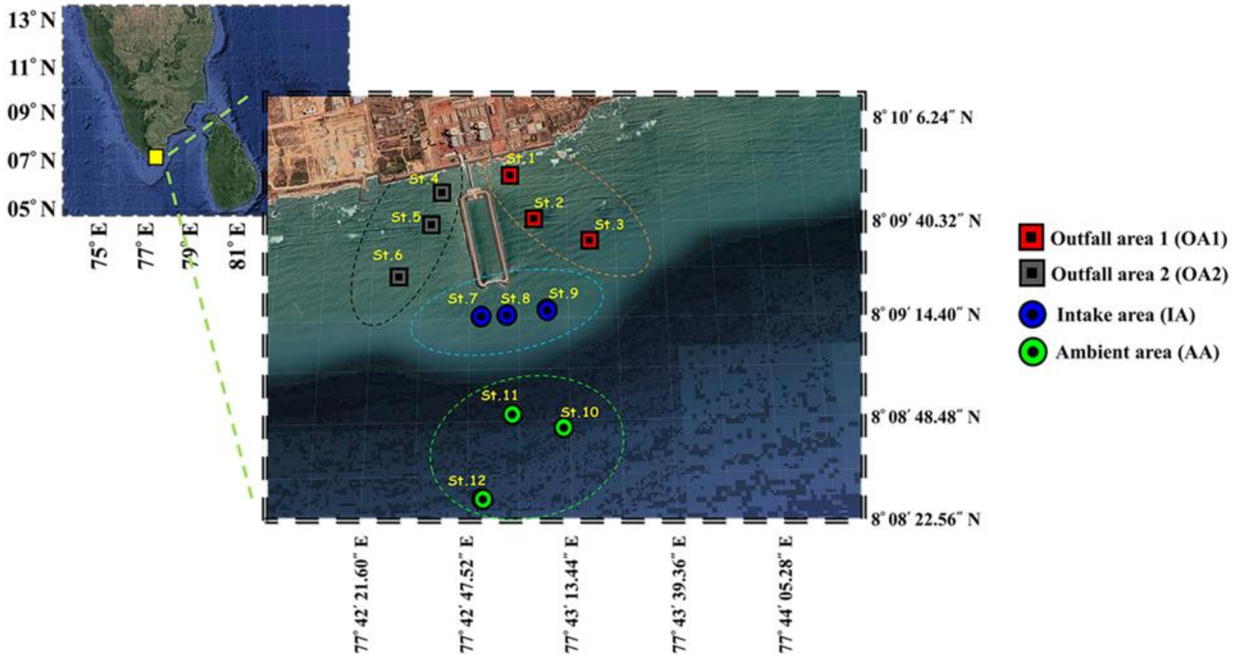

Supplementary Figure S2. Temperature profile and concentrations of inorganic nutrients such as nitrite (NO_2), nitrate (NO_3), soluble reactive phosphorus (PO_4) and ammonium (NH_4) in the control (IA and AA) and thermal discharge-impacted (OA1 and OA2) areas. ($\Delta T1$ = temperature difference between control and thermal discharge area OA1, $\Delta T2$ = temperature difference between control and area OA2).

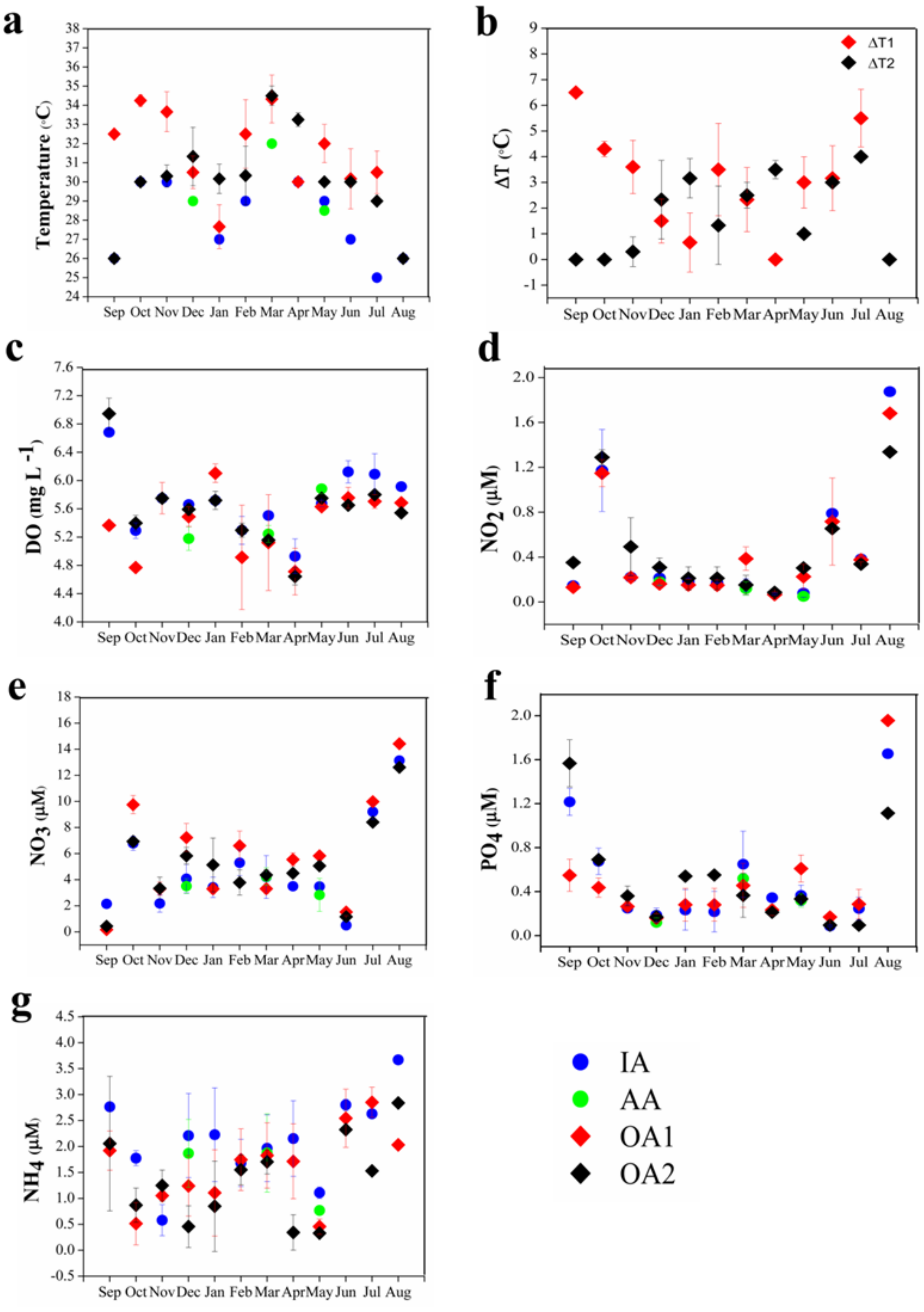

Supplementary Figure S3. Rarefaction curves of observed bacterioplankton OTUs (defined at 97% sequence similarity) and obtained V3-V4 hypervariable region sequences of individual seawater samples.

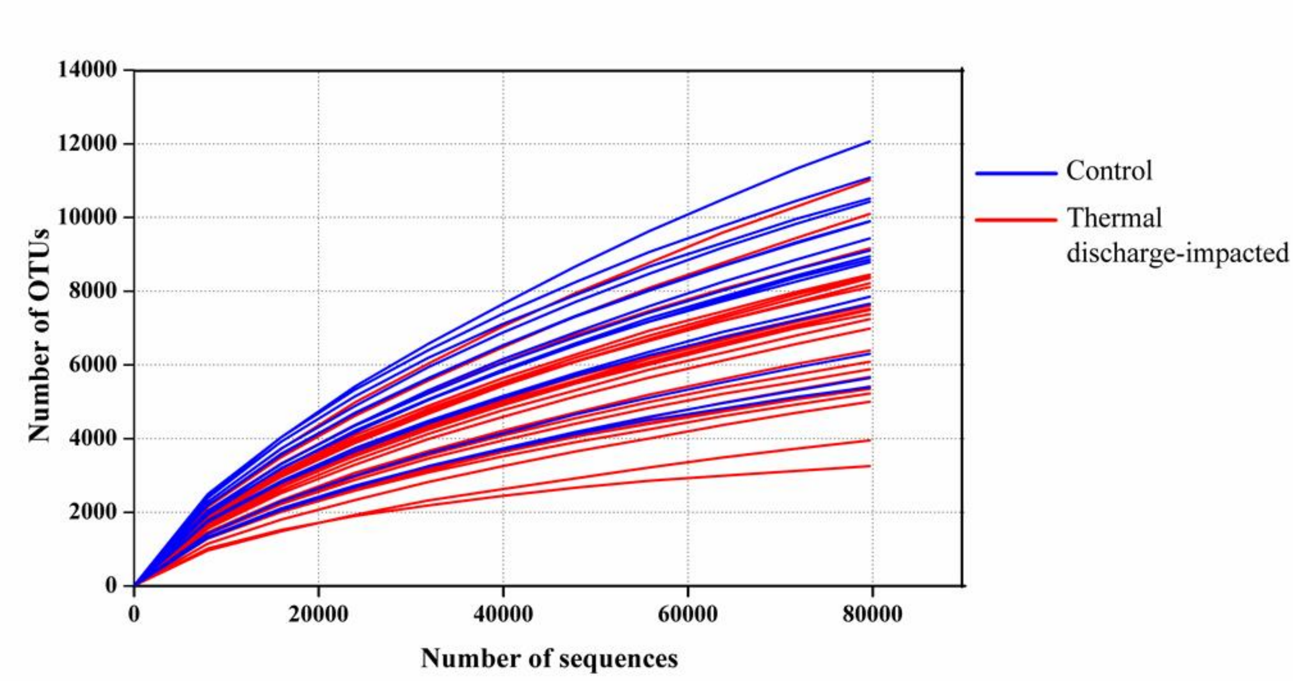

Supplementary Figure S4. Heatmap analysis of bacterioplankton communities (at phylum level) for the seawater samples collected during (a) September-2016, (b) October-2016, (c) November-2016, (d) December-2016, (e) January-2017, (f) February-2017, (g) March-2017, (h) April-2017, (i) May-2017, (j) June-2017, (k) July-2017, (l) August-2017 and composite (m).

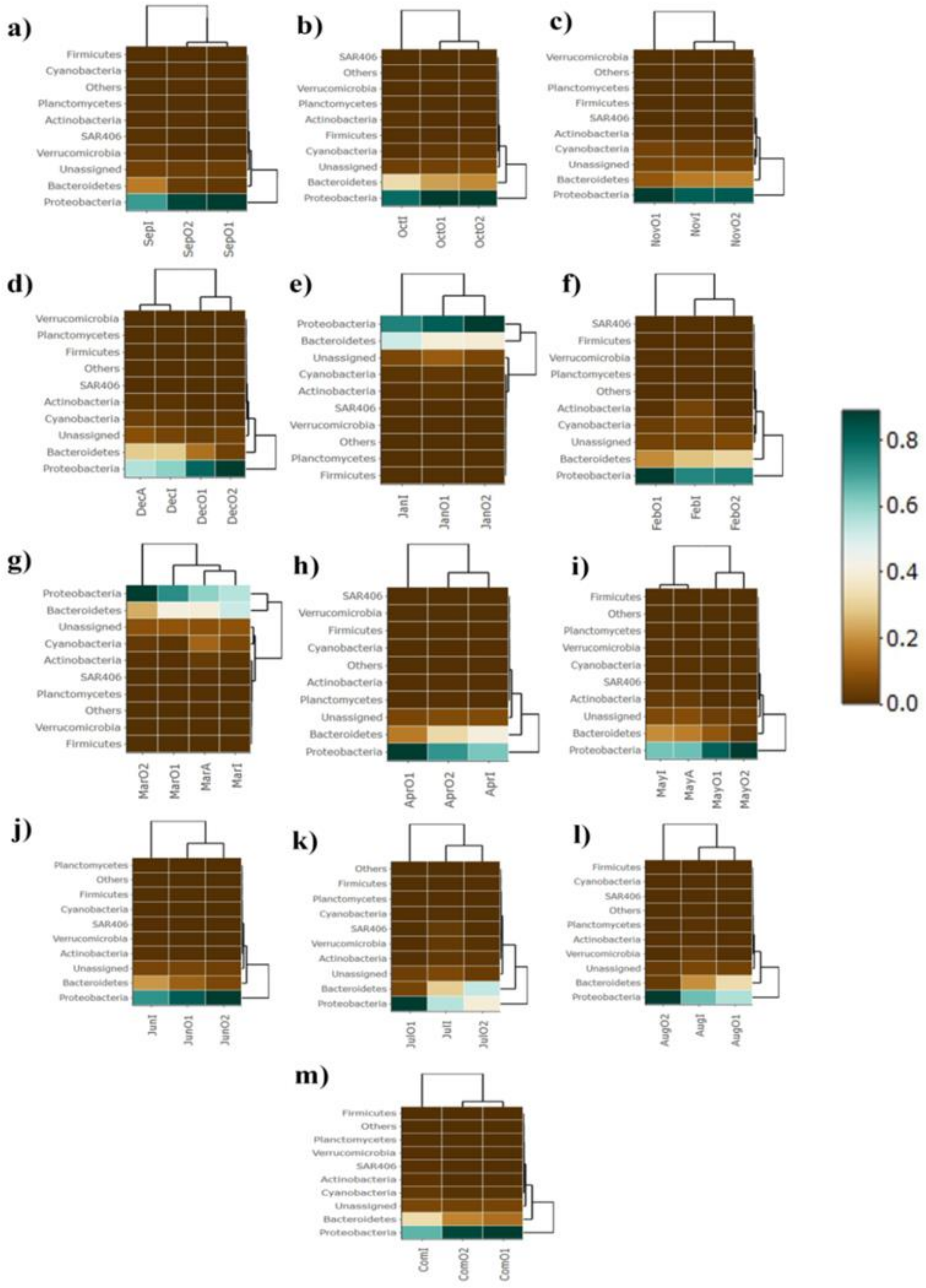

Supplementary Figure S5. Comparison of bacterial community composition between control and thermal discharge-impacted sample groups at genus level. The significantly difference in mean proportion of each bacterial genus was determined in STAMP software using Welch's t-test and displayed as extended error bar (two sided).

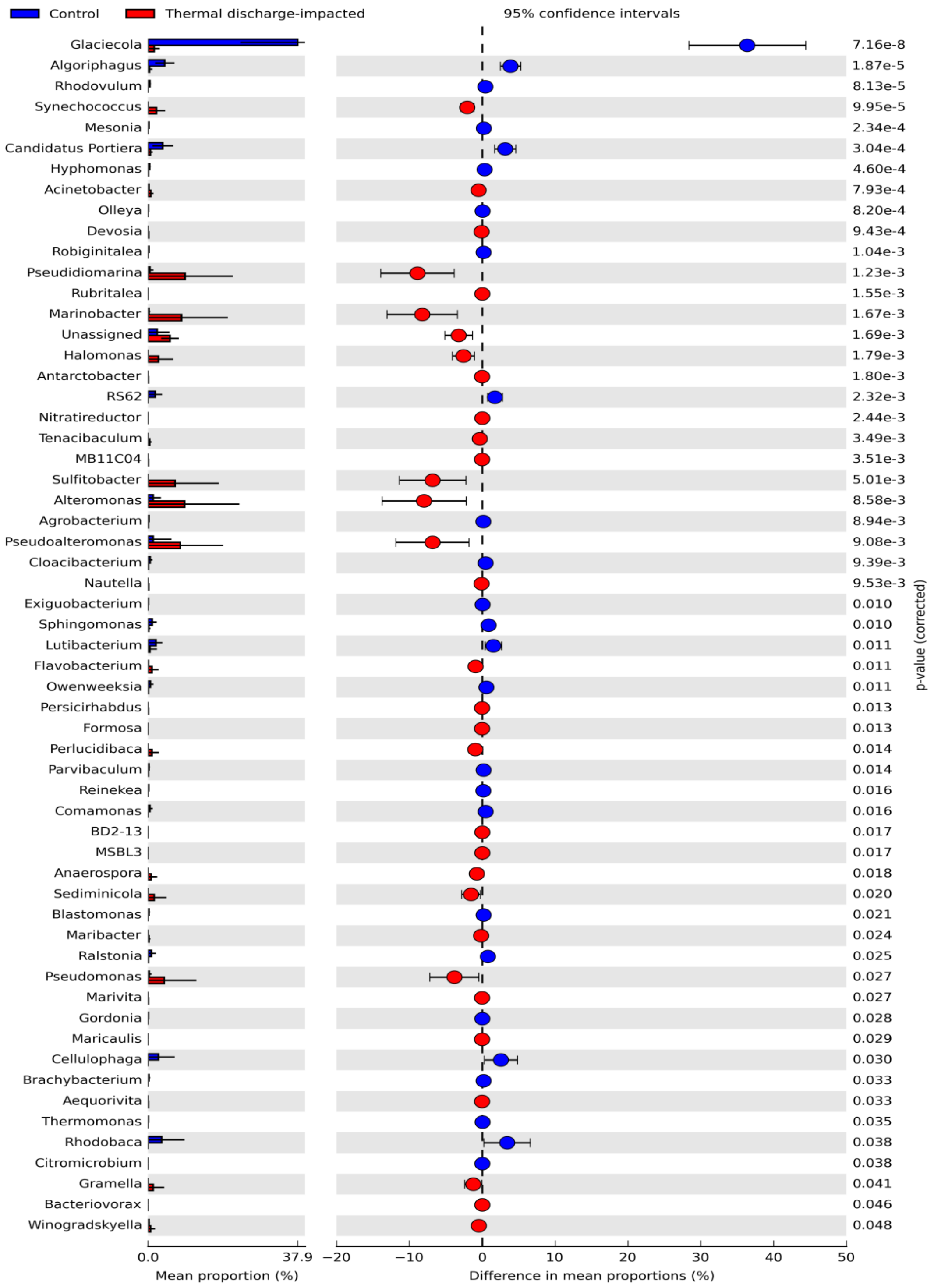

Supplement: Supplementary file 1 — Supplementary Information. [file 41598_2021_96969_MOESM1_ESM.pdf]
